# Study of Strain-Hardening Behaviour of Fibre-Reinforced Alkali-Activated Fly Ash Cement

**DOI:** 10.3390/ma12234015

**Published:** 2019-12-03

**Authors:** Hyuk Lee, Vanissorn Vimonsatit, Priyan Mendis, Ayman Nassif

**Affiliations:** 1 School of Civil and Mechanical Engineering, Curtin University, Perth, WA 6102, Australia; V.Vimonsatit@curtin.edu.au; 2 Department of Infrastructure Engineering, University of Melbourne, Parkville, VIC 3010, Australia; pamendis@unimelb.edu.au; 3 School of Civil Engineering and Surveying, The University of Portsmouth, Portsmouth, Hampshire PO1 2UP, UK; ayman.nassif@port.ac.uk

**Keywords:** fibre reinforced, alkali-activated, strain hardening

## Abstract

This paper presents a study of parameters affecting the fibre pull out capacity and strain-hardening behaviour of fibre-reinforced alkali-activated cement composite (AAC). Fly ash is a common aluminosilicate source in AAC and was used in this study to create fly ash based AAC. Based on a numerical study using Taguchi’s design of experiment (DOE) approach, the effect of parameters on the fibre pull out capacity was identified. The fibre pull out force between the AAC matrix and the fibre depends greatly on the fibre diameter and embedded length. The fibre pull out test was conducted on alkali-activated cement with a capacity in a range of 0.8 to 1.0 MPa. The strain-hardening behaviour of alkali-activated cement was determined based on its compressive and flexural strengths. While achieving the strain-hardening behaviour of the AAC composite, the compressive strength decreases, and fine materials in the composite contribute to decreasing in the flexural strength and strain capacity. The composite critical energy release rate in AAC matrix was determined to be approximately 0.01 kJ/m${}^{2}$ based on a nanoindentation approach. The results of the flexural performance indicate that the critical energy release rate of alkali-activated cement matrix should be less than 0.01 kJ/m${}^{2}$ to achieve the strain-hardening behaviour.

## 1. Introduction

Alkali-activated cement (AAC) is a potential cementitious system to be introduced as an alternative cement [1,2]. AAC-based concrete exhibits a variety of advantageous properties and characteristics, such as high strength, low shrinkage, fast setting time, good acid and fire resistance, and low thermal conductivity. A highly concentrated alkali hydroxide solution or silicate solution that reacts with solid aluminosilicate produces synthetic alkali aluminosilicate materials [2]. These materials are classified as polymers because their structures are large molecules formed by number of group of smaller molecule [3]. The form of one such polymer is the product of the reaction of an alkali solution and source materials, such as fly ash—which is rich in aluminosilicate and includes organic minerals, such as kaolinite and inorganic material [4].

Cementitious materials, such as mortar and concrete, generally show brittle behaviour. Historically, traditional reinforcement in concrete was in the form of continuous reinforcing bars, which should be in an appropriate location to resist the imposed tensile and shear stresses. In a fibre reinforced cementitious composite, fibres are discontinuous and are randomly distributed throughout the cementitious matrix. They tend to be more closely located than conventional reinforcing bars, and are therefore better at controlling cracking. High performance fibre reinforced cementitious composite (HPFRCC) is a type of material that exhibits a pseudo strain-hardening characteristic under uniaxial tensile stress in fibre reinforced cementitious composites. The “high performance” refers to the quality a fibre reinforced cementitious composite based on the shape of its stress–strain curve in fibre orientations [5]. HPFRCC can be generally classified by composite mechanics, energy, and numerical approaches. One way to define the condition to accomplish strain hardening behaviour is that post-cracking strength of the composite is higher than its cracking strength. It is, therefore, necessary to understand some important parameters which are related to the shape of the stress–strain relationship of HPFRCC [6]. Several research works [7,8,9,10] reported strain-hardening behaviour of cementitious materials; however, the performance of fibre reinforced AAC composite is still an enigma. Fly ash is a common aluminosilicate source in AAC; therefore, in this research, an investigation was carried out on the affects of fibre contents in alkali-activated fly ash cement (AAFA) composites. The experimental works were to determine fibre interfacial strength in AAFA matrices, and the numerical analysis approach using Taguchi’s DOE method was to determine the effect of the parameters on AAFA matrix. Furthermore, the compressive strength development and the strain-hardening behaviour of AAFA composites were studied to examine the structural performance under compression and flexure.

## 2. Materials and Methods

**Figure 1:**
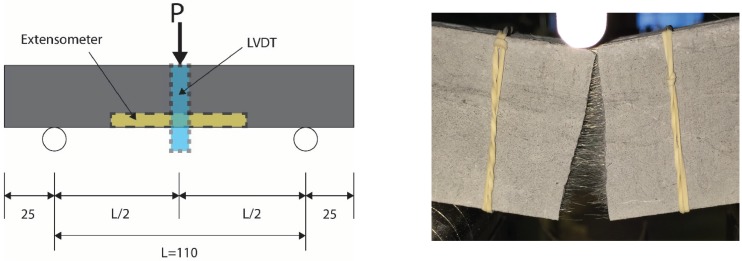
Configuration of flexural performance.

Class F (low calcium) fly ash available locally in Australia was used to prepare AAFA matrices. The summary of chemical compositions of fly ash is presented in Table 1. The specimens were cast in 25 mm cubic moulds for the compressive strength test, which was modified based on ASTM C109, and in prismatic specimens of 160 × 40 × 40 mm for a 3-point flexural performance test according to ASTM C78 as shown in Figure 1. The monofilament polyvinyl alcohol (PVA) fibre was used in this research; its diameter and length are 38 μm and 8 mm, respectively. PVA fibre has high chemical bond strength due to the hydrophilic nature and highly alkali resistant characteristic. The tensile strength and elastic modulus of PVA fibre were reported as 1600 MPa and 40 GPa, respectively.

The specimens were cured for 24 h at 60 ${}^{\circ }$C which is a common curing temperature for AAC [3,11,12]. After that, the specimens were placed in a curing room at 23 ${}^{\circ }$C ± 3 until testing. The compressive strength test was conducted at 7, 14, and 28 days of curing age while the flexural test was conducted on day 28 of curing. Each test was repeated on six samples. The selected mixing proportion is the process of choosing suitable fibre volume fraction of AAFA mixtures, as shown in Table 2; there were two main groups, with and without silica fume, and with varying fibre volume fraction in AAFA mixtures. The liquid to solid ratio and the content of superplasticiser were 0.5 and 0.02%, respectively.

**Figure 2:**
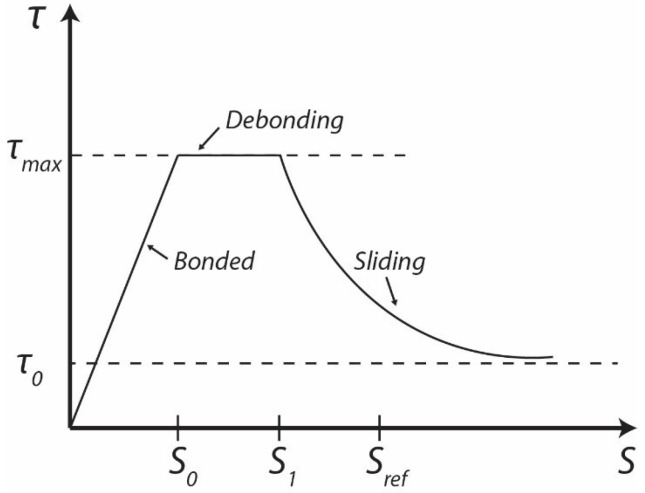
Idealised interface law in three stages of single fibre pull out (adopted after [16]).

It is important to note that interface’s properties between fibre and matrix significantly influence the performance of a composite. The interfacial properties are also important in the fracture mechanism and the fracture toughness of the composite. The failure process in a composite material when a crack propagates is complex and involves matrix cracking. The bonding strength between fibre and matrix is to be considered as a source of energy dissipation of HPFRCC. The single fibre pull out test is the most common method to understanding the interfacial strength. Generally, the fibre pull out has three stages during debonding [13,14,15], as shown in Figure 2. Each stage of a single fibre pull out test can be expressed by:

- The first stage, $S_{0}$: the fibre and matrix is bonded until reaching the maximum interfacial bond strength $\tau_{max}$;
- The second stage, $S_{0}-S_{1}$: a crack propagation could occur along the interface between the fibre and matrix which leads to complete debonding;
- The third stage, $S_{1}-S_{ref}$: fibre is pulled out from the matrix and starts to slip;
- Thus, the maximum pull out force is the most important parameter of HPFRCC, which can present maximum interfacial bond strength.

**Figure 3:**
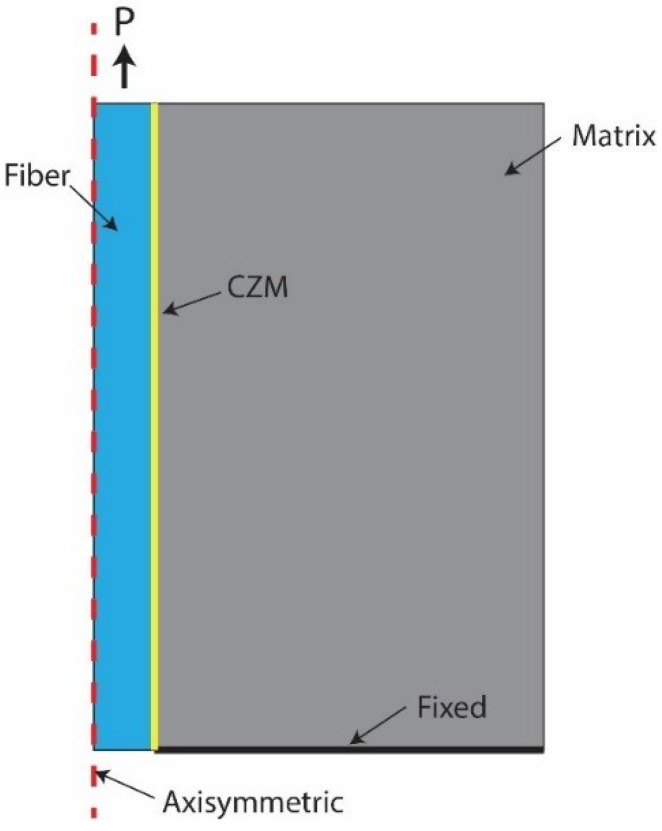
Configuration of single fibre pull out simulation without an inclined angle.

**Figure 4:**
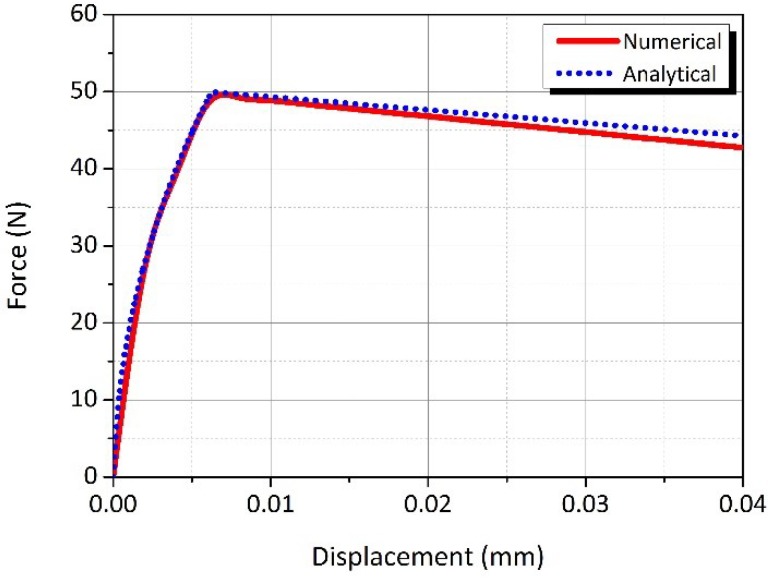
Validation of finite element (FE) model with the analytical model by Zhan et al. [16].

A numerical study for the behaviour of single fibre pull out was carried using commercial finite element (FE) software package ANSYS [17]. A 2-D axisymmetric model was employed for the simulation of the single fibre pull out process. In the developed model, a PVA fibre with a radius $R_{f}$ was embedded at the centre of the cylindrical matrix, and $L_{d}$ was the total embedded length of the fibre. The bottom of the model was constrained in both radial and axial directions. The interfacial properties were modelled using the bilinear cohesive zone model (CZM) in mode II, which was established by fracture mechanic models, such as the interface traction and separation. The relationship between normal critical energy $G_{cn}$ and tangential critical energy $G_{ct}$ can be expressed by the maximum normal contact stress $\sigma_{max}$, the maximum tangential contact stress $\tau_{max}$, the complete normal displacement $\delta_{n}$, and the complete tangential displacement $\delta_{t}$ [17]. Figure 3 presents the model of the FE single fibre pull out test with the fibre and matrix model which were meshed with 122,406 six node quadrilateral elements. The model was analysed using a non-linear geometrical method with convergent displacement control. To confirm the validity of the FE analysis of the single fibre pull out, an analytical fibre pull out test was conducted. An interfacial friction law for the slip mechanism between the fibre and the matrix has been investigated by several authors [16,18,19]. For an analytical fibre pull out, a proposed model by Zhan et al. [16], which was based on the interfacial law that could capture the major mechanism involved in various situations, was used to obtain the fibre pull out force. The results of the analytical and the FE analyses of the single fibre pull out model were overall in good agreement, with around 2% difference, as illustrated in Figure 4. Thus, the FE simulation can be used for investigating the interfacial behaviour between the fibre and the AAFA matrix.

Taguchi’s DOE approach with eight parameters and three levels of test variables were selected in accordance to the literature [16,18,20,21,22,23], as shown in Table 3. The standard L${}_{27}$ (3${}^{13}$) orthogonal array was used in accordance to these parameters, and the detail of L${}_{27}$ orthogonal array is shown in Table 4.

## 3. Results and Discussion

### 3.1. Single Fibre Pull Out

**Figure 5:**
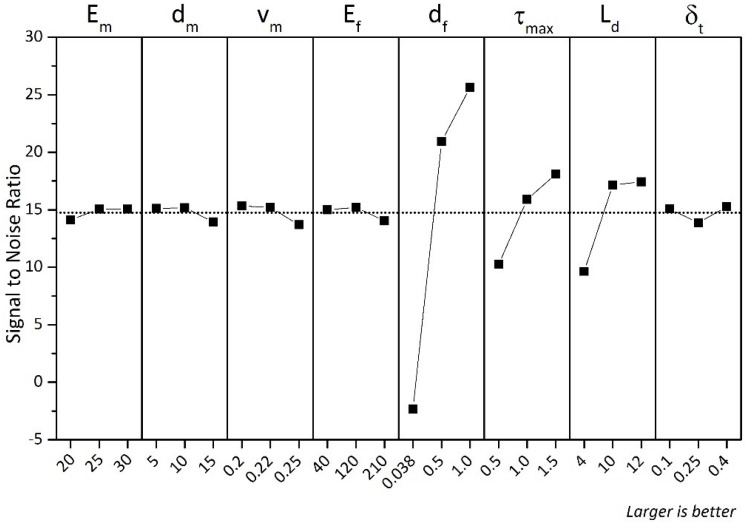
Signal to noise ratio of single fibre pull out.

Based on the Taguchi’s DOE approach, a statistical signal to noise (S/N) ratio analysis was performed to determine the effect of these parameters on the maximum fibre pull out force $P_{max}$, as illustrated in Table 5 and Figure 5. The S/N ratio shows that the diameter of the fibre has the most effect on the fibre pull out force. The elastic modulus of the fibre and the matrix has a minor effect on the pull out force. A further analysis of the single fibre pull out behaviour was done using analysis of variance (ANOVA) and the results indicate that the contribution of the fibre diameter on pull out force is 44.69% of the total contribution factors. The overall results are presented in Table 6. It can be observed that increasing the elastic modulus of the matrix, the diameter of the fibre, the tangential traction, and the embedded length of the fibre results in increasing the pull out force. The contributions of the elastic modulus of the matrix, the tangential traction and the embedded length of the fibre on the pull out force are 14.48%, 8.92%, and 9.47%, respectively. At the same time, increasing the Poisson’s ratio results in decreasing in the pull out force but the contribution is minor. The contributions of the diameter of the fibre, Poison’s ratio, the elastic modulus of matrix, and complete tangential displacement on the pull out force are fairly similar at about 2.5%.

The failure process in a composite material when a crack propagates is complex and involves matrix cracking. The bonding strength between fibre and matrix is to be considered as a source of energy dissipation. Thus, a single fibre pull out test of PVA fibre conducted with AAFA paste matrix (l/s = 0.6) was also conducted with OPC paste matrix (w/c = 0.3) to compare the interfacial bonding strength between AAFA and OPC matrices. The embedded length ($L_{d}$) of the fibre was around 4 mm which is half of the total length of the fibre, and the diameter of fibre ($d_{f}$) was 38 μm. Assuming uniform bonding, the maximum interfacial bonding stre. The results of the single fibre pull out test of AAFA and OPC matrices show that the pull out force is similar. A comparison of the maximum pull out force between the numerical and experimental results are presented in Table 7. The input parameters such as elastic modulus and Poisson’s ratio of the FE model were adopted from the authors’ previous work [24]. The comparison of the maximum pull out force between the numerical and experimental results are in good agreement, thus, validating the numerical analysis of the single fibre pull out with Taguchi’s DOE.

### 3.2. Compressive Strength

**Figure 6:**
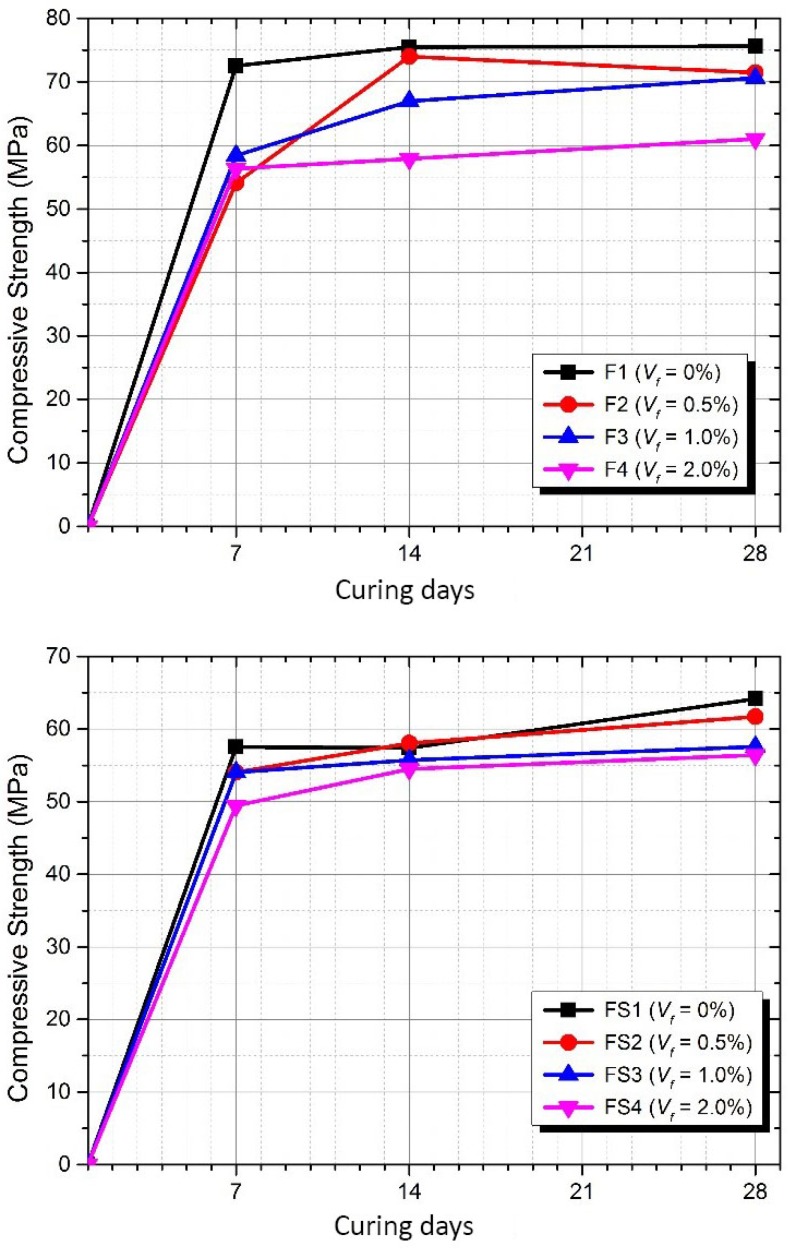
Compressive strength developments of Group A (**top**) and Group B (**bottom**).

Figure 6 shows the average compressive strength development from 7 to 28 days of curing age in each composite. It can be seen that the compressive strength of AAFA composite generally decreases with increasing fibre volume fraction ratio. Also, it was observed that the compressive strength development was not significantly increased by the fibre volume fraction ratio in F2 mixture, which exhibited a high rate of compressive strength development between 7 to 14 days of curing ages. The test results indicate that the compressive strength development is not significantly affected by the fibre volume fraction ratio.

**Figure 7:**
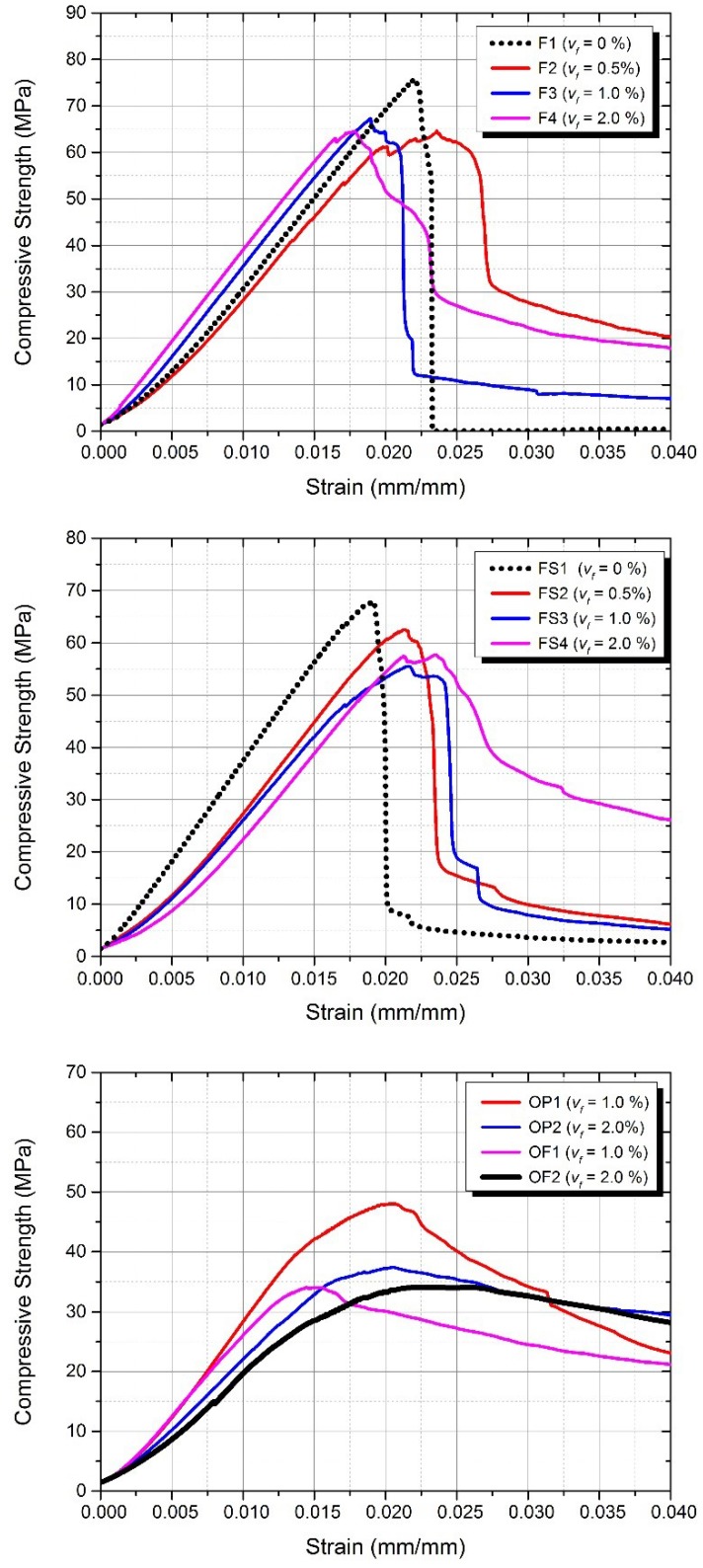
Compressive stress–strain curves of Group A (**top**) and Group B (**bottom**).

The behaviour and the ultimate compressive failure mode of AAFA composites are shown in Figure 7. It is known that PVA fibre matrix can exhibit ductile behaviour after reaching its compressive strength because of the transverse confinement effect of the PVA fibre, while normal AAFA mixtures without PVA fibre (F1 and FS1 mixtures) present a significant decrease in stress after reaching their ultimate compressive strength. However, OPC composites (w/c = 0.4) have more ductile behaviour after reaching their ultimate compressive strength than that of AAFA composites, as shown in Figure 7. It can also be seen that the post-peak behaviour depends on the fibre content; those mixes with the same fibre content show similar post-peak behaviour. It can be seen that the compressive strain is not significantly affected by the fibre volume fraction ratio. Further, the compressive strain corresponding to the compressive strength is not meaningfully affected. However, the compressive strength generally decreases with increasing fibre volume fraction ratio and the content of the added silica fume in AAFA composites led to lower compressive strength. In a previous research study [24], it was observed that silica fume in AAFA matrix contributed to a significant decrease in the compressive strength due to a decrease in the cohesion of the reaction products.

### 3.3. Flexural Performance

**Figure 8:**
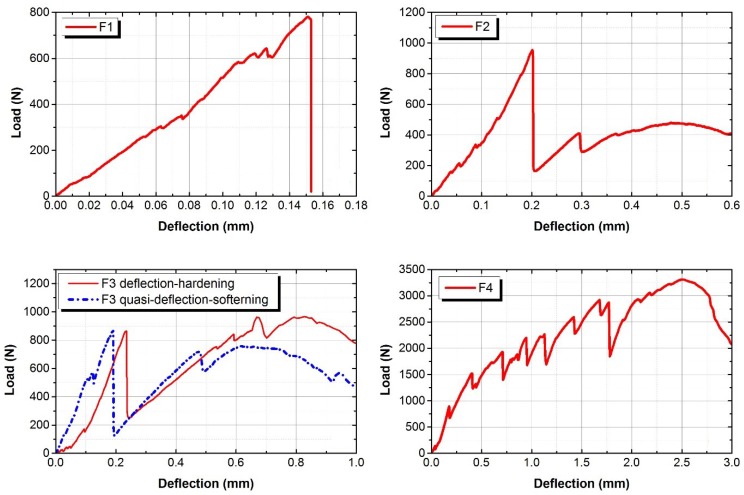
Flexural behaviour of Group A.

**Figure 9:**
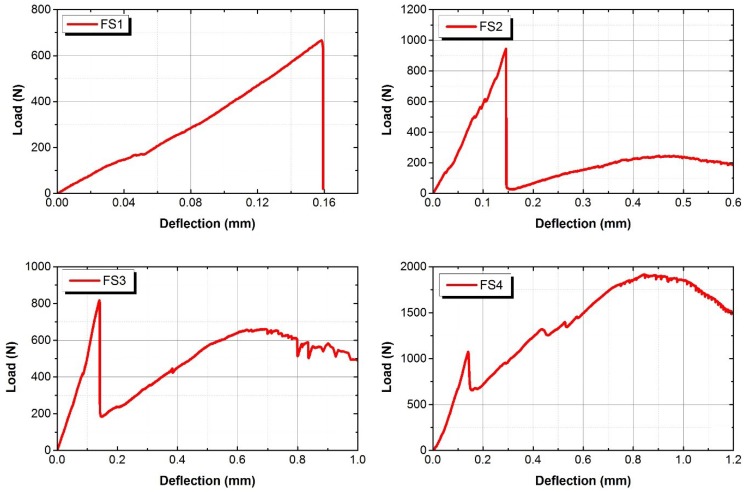
Flexural behaviour of Group B.

**Figure 10:**
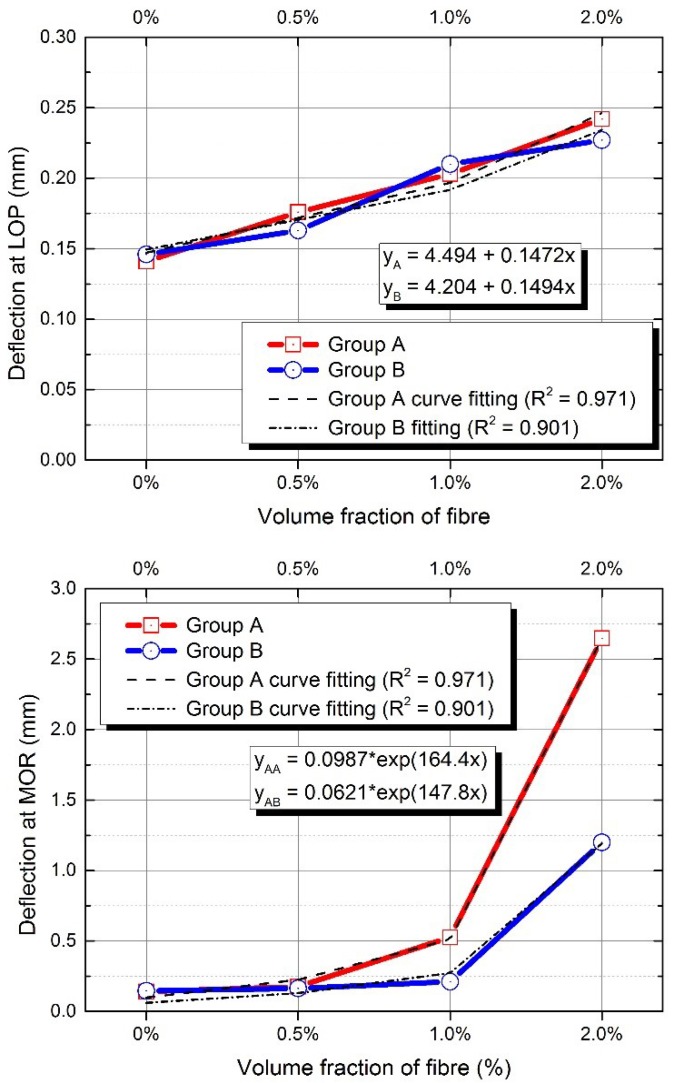
Effect of fibre volume fraction on deflection at limit of proportionality (LOP) (**top**) and modulus of rupture (MOR) (**bottom**).

The flexural behaviour of composites will exhibit deflection-hardening, or softening behaviour after, first, cracking. The first cracking point of the composite is defined as limit of proportionality (LOP), and the maximum equivalent flexural strength point of the composite is defined as modulus of rupture (MOR) [25]. The flexural behaviours of AAFA composites are shown in Figure 8 and Figure 9. The flexural performance of F1 mixture shows a typical form of deflection-softening behaviour; F2 mixture shows quasi-deflection-softening behaviour; and F4 mixture shows deflection-hardening behaviour. However, for F3 mixture, some of the specimens had complex behaviours, which were deflection-hardening and quasi-deflection-softening behaviours. The maximum loading capacity of F4 mixture was observed to be about 74% greater than that of other mixtures, and the deflection capacity of F4 mixtures was also observed to be greater than that of F1, F2, and F3 mixtures. Similarly, the flexural performance of FS1, FS2, and FS3 mixtures showed typical deflection-softening behaviours, while F4 mixture presented deflection-hardening behaviour. The maximum loading and deflection capacity of F4 mixture were found to be around 65% and 85% greater than the maximum loading and the deflection capacities, respectively, of other mixtures. As the volume fraction ratio of fibre in the AAFA composite increased from 0% to 2.0%, the effects of the fibre volume fraction ratio on the deflection capacities of different mixtures of AAFA composites were plotted in Figure 10. There results show an increasing trend of the deflection capacity at LOP as the linear relationship, and an increasing trend of the deflection capacity at MOR, as the exponential relationship. The improvement of deflection at MOR in Group A was observed to have much higher deflection capacity than that of Group B. Li et al. [6,22,26] reported that adding fine aggregates in OPC composite could improve the pseudo-strain hardening behaviour. In AAFA composite, however, adding fine aggregates (SF) in this case does not improve the flexural deflection and strength capacity. The flexural behaviour of AAFA composite with SF as added fine aggregates shows a decrease in the flexural strength and no improvement in the flexural deflection and strain capacity.

**Figure 11:**
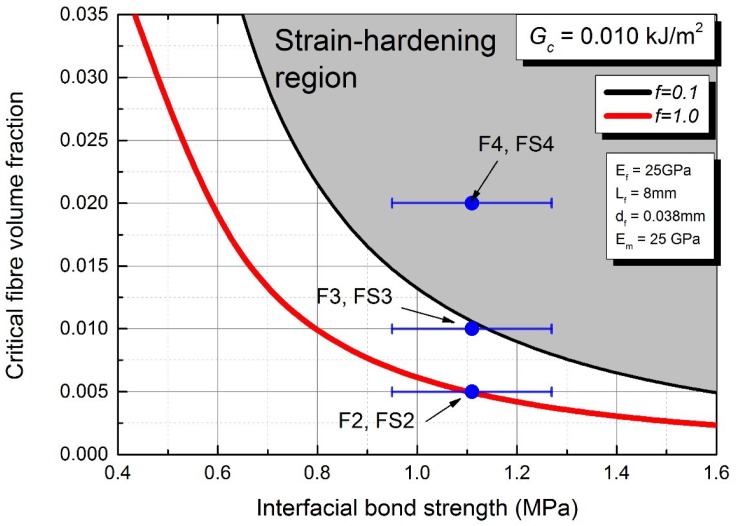
Critical volume fraction against interfacial bond strength.

According to the literature [5,27,28], the tensile and compressive behaviour of a composite material strongly influence the flexural performance. Also, the strain-hardening behaviour in tension leads to a deflection-hardening behaviour when the flexural behaviour of the composites is strongly associated with its tensile characteristic [29]. Thus, the results of the flexural performance obtained in this research could be related with the tensile behaviour of AAFA composites. Based on the theoretical discussions by several researchers [5,6,7,9,22,26,30], the critical energy release rate $G_{c}$ and the interfacial bond strength τ of the composites are important parameters to be considered in a design of the composite’s matrix to achieve the strain-hardening behaviour of the composite. In addition, the matrix properties, such as elastic modulus and fracture toughness, which are linked to the composite’s critical energy release rate $G_{c}$, are affected by several parameters [7,22]. Using nanoindentation data, the composite critical energy release rate $G_{c}$ of AAFA matrix was found to be 0.010 kJ/m${}^{2}$. Based on the fracture toughness and the elastic modulus of AAFA matrix [24], the interfacial bond strength of AAFA composite was plotted against the critical fibre volume fraction ratio and the corresponding strain-hardening behaviour with the snubbing coefficient *f*, which is in term of the inclining angle between fibre and matrix, as illustrated in Figure 11. It can be seen that with 2.0% of the fibre volume fraction ratio in the AAFA composites, F4 and FS4 mixtures are in the region of strain-hardening, whereas, with less than 0.5% of the fibre volume fraction ratio, F2 and FS2 are not in the region of strain-hardening. It can also be noticed that with 1.0% of the fibre volume fraction ratio, F3 and FS3 mixtures are partly in the region of strain-hardening with other parts falling in the region of strain-hardening. This is consistent with F3 mixture, which shows a combination of strain-hardening and quasi-strain-hardening behaviour.

## 4. Conclusions

The experimental and theoretical studies of parameters affecting the fibre pull out capacity and the strain-hardening behaviour of AAFA composites have been presented. Based on the results obtained in this research, the following conclusion can be drawn:

- The interfacial bond strength between the fibre and the AAFA matrix was determined to be in a range of 0.8 to 1.0 MPa. A numerical analysis coupled with a statistical analysis tool shows that an increase in the fibre diameter and embedded length would increase the interfacial bond strength.
- The strain corresponding to the compressive strength is not significantly affected by the fibre volume fraction ratio. However, while achieving the strain-hardening behaviour of the AAFA composites, the compressive strength decreased. In addition, using silica fume as a fine material in AAFA composite is not suitable as it decreases the flexural strength and strain capacity of the composite.
- The critical energy release rate $G_{c}$ of AAFA matrix determined from the indentation fracture toughness was approximately 0.01 kJ/m${}^{2}$. The results of the flexural behaviour showed the relationship between the strain-hardening behaviour of AAFA composite and the indentation $G_{c}$.
- For a mix design of AAFA matrix, it is recommended that $G_{c}$ should be less than 0.01 kJ/m${}^{2}$. It is theoretically impossible to achieve the strain-hardening behaviour when $G_{c}$ is more than 0.015 kJ/m${}^{2}$.

## Figures and Tables

**Table 1:** Chemical composition of low calcium fly ash (wt. %).

SiO${}_{2}$	Al${}_{2}$O${}_{3}$	CaO	Fe${}_{2}$O${}_{3}$	K${}_{2}$O
65.9	24.0	1.59	2.87	1.44

**Table 2:** Mix proportion.

Group	Index	Fly Ash	Silica Fume	PVA Fiber *
A	F1	1	-	-
	F2	1	-	0.5%
	F3	1	-	1.0%
	F4	1	-	2.0%
B	FS1	1	0.2%	-
	FS2	1	0.2%	0.5%
	FS3	1	0.2%	1.0%
	FS4	1	0.2%	2.0%

* by volume fraction.

**Table 3:** Variation parameters and levels.

Parameter	Level 1	Level 2	Level 3
Elastic modulus of matrix, $E_{m}$ (GPa)	20	25	30
Diameter of matrix, $d_{m}$ (mm)	5	10	15
Poisson’s ratio of matrix $\nu_{m}$	0.2	0.22	0.25
Elastic modulus of fibre $E_{f}$ (GPa)	40	120	210
Diameter of fibre, $d_{f}$ (mm)	0.038	0.5	1
Fibre embedded length, $L_{d}$ (mm)	4	10	12
Maximum tangential traction, $\tau_{t}^{max}$ (MPa)	0.5	1	1.5
Complete tangential displacement, $\delta_{t}$ (mm)	0.1	0.25	0.4

**Table 4:** Standard L${}_{27}$ orthogonal array.

No.	$E_{m}$	$d_{m}$	$\nu_{m}$	$E_{f}$	$d_{f}$	$L_{d}$	$\tau_{t}^{\max}$	$\delta_{\max}$
1	1	1	1	1	1	1	1	1
2	1	1	1	1	2	2	2	2
3	1	1	1	1	3	3	3	3
4	1	2	2	2	1	1	1	2
5	1	2	2	2	2	2	2	3
6	1	2	2	2	3	3	3	1
7	1	3	3	3	1	1	1	3
8	1	3	3	3	2	2	2	1
9	1	3	3	3	3	3	3	2
10	2	1	2	3	1	2	3	1
11	2	1	2	3	2	3	1	2
12	2	1	2	3	3	1	2	3
13	2	2	2	3	1	1	3	2
14	2	2	3	1	2	3	1	3
15	2	2	3	1	3	1	2	1
16	2	3	1	2	1	2	3	3
17	2	3	1	2	1	2	3	3
18	2	3	1	2	3	1	2	2
19	3	1	3	2	1	3	2	1
20	3	1	3	2	1	3	3	2
21	3	1	3	2	3	2	1	3
22	3	2	1	3	1	3	2	2
23	3	2	1	3	2	1	3	3
24	3	2	1	3	3	2	1	1
25	3	3	2	1	1	3	2	3
26	3	3	2	1	2	1	3	1
27	3	3	2	1	3	2	1	2

**Table 5:** Numerical studies of single fibre pull out with Taguchi’s DOE.

No.	$P_{\max}$ (N)	No.	$P_{\max}$ (N)	No.	$P_{\max}$ (N)
1	0.23	10	1.27	19	1.43
2	15.51	11	9.42	20	9.41
3	56.09	12	15.71	21	12.57
4	0.24	13	1.09	22	1.67
5	15.67	14	9.41	23	9.41
6	55.95	15	15.59	24	12.56
7	0.24	16	1.36	25	1.51
8	15.61	17	9.40	26	9.19
9	14.61	18	15.69	27	12.56

**Table 6:** Analysis of Variance of fibre pull out force.

Source	DF ${}^{a}$	SS ${}^{b}$	MS ${}^{c}$	Contribution %
$E_{m}$	2	737.6	368.8	14.48
$d_{m}$	2	127.2	63.6	2.50
$\nu_{m}$	2	129.3	64.7	2.54
$E_{f}$	2	124.0	61.9	2.43
$d_{f}$	2	2276.5	1138.3	44.69
$L_{d}$	2	454.4	227.2	8.92
$\tau_{t}^{max}$	2	482.5	241.2	9.47
$\delta_{max}$	2	126.7	63.4	2.49
Error	10	653.8	63.6	12.48

${}^{a}$ degree of freedom; ${}^{b}$ sum of squares; ${}^{c}$ mean of squares.

**Table 7:** Maximum pull out force in the finite element and the experimental results.

Matrix	FEM	Experimental	Ratio
OPC	0.482	0.480	1.001
AAFA	0.530	0.530	1.000
